# A Vascularized Multilayer Chip Reveals Shear Stress-Induced Angiogenesis in Diverse Fluid Conditions

**DOI:** 10.34133/cbsystems.0207

**Published:** 2025-02-28

**Authors:** Tao Yue, Huiying Yang, Yue Wang, Ning Jiang, Hongze Yin, Xiaoqi Lu, Na Liu, Yichun Xu

**Affiliations:** ^1^ School of Mechatronic Engineering and Automation, Shanghai University, Shanghai, China.; ^2^ School of Future Technology, Shanghai University, Shanghai, China.; ^3^ Shanghai Key Laboratory of Intelligent Manufacturing and Robotics, Shanghai University, Shanghai, China.; ^4^ Shanghai Institute of Intelligent Science and Technology, Tongji University, Shanghai, China.; ^5^ National Engineering Research Center for Biochip at Shanghai, Shanghai, China.; ^6^ Shanghai Biochip Corporation (SBC), Shanghai, China.

## Abstract

Tissues larger than 400 μm in size lacking microvascular networks cannot survive for long periods of time in vitro. The development of microfluidic technology provides an efficient research tool for constructing microvascular models in vitro. However, traditional single-layer microfluidic chips faced the limitation of spatial layout and could not provide diverse fluidic environments within a single chip. In this paper, we present a novel microfluidic chip design with a 3-layer configuration that utilizes a polycarbonate (PC) porous membrane to separate the culture fluid channels from the tissue chambers, featuring flexibly designable multitissue chambers. PC porous membranes act as the capillary in the vertical direction, enabling precise hydrogel patterning and successfully constructing a microfluidic environment suitable for microvascular tissue growth. The chip demonstrates the ability to build microvascular networks of different shapes such as triangle, rectangle, and inverted triangle on a single chip for more than 10 days. The microvascular networks cultured for 12 days were successfully perfused with 70-kDa fluorescein isothiocyanate, which indicated that the generated networks had good barrier properties. A correlation between tissue chamber shape and shear stress was demonstrated using COMSOL, and a preliminary validation of the flow direction of interstitial flow and the important effect of shear stress on microvascular growth was demonstrated by vascularization experiments. This flexible and scalable design is ideal for culturing multiple vascularized organ tissues on a single microfluidic chip, as well as for studying the effects of different fluidic factors on microvascular growth.

## Introduction

Microvessels are small vascular networks that connect arteries and veins, facilitating microcirculation among tissues and organs by delivering oxygen, nutrients, and metabolites [1]. Most cells in vivo are located within 200 μm of microvessels [2]. To study the physiological and pathological features associated with the development and formation of microvasculature, it is important to establish models in vitro associated with microvascular tissues [3]. However, simulating complex organ models in vivo with conventional 2-dimensional cultures is challenging. Animal models are also highly debated due to ethical issues [4]. Microfluidics, with its ability to highly simulate the microenvironment in vivo, provides an effective solution for constructing microvascular tissues in vitro [5–7].

There are 2 main strategies for constructing microvessels in vitro, including the predesigned vascularization method and the self-organized vascularization method [8]. In the predesigned vascularization method, microchannels were fabricated using micromachining techniques and the microvascular lumen was arranged based on the shape of these microchannels [9–13]. However, this method was limited in its ability to replicate the complex interactions and dynamics of real microvascular tissues, potentially resulting in a less representative model. Conversely, the self-organized vascularization method involved seeding cells into the extracellular matrix (ECM) and controlling the microenvironment, including fluid flow and chemical factors, within a microfluidic chip [14–17]. This approach induced the spontaneous formation and construction of microvascular networks that closely approximated the intricacies and physiological environment in the human body [18]. By more accurately replicating these conditions, this method offered a more physiologically relevant model for studying microvessels in vitro.

Many current microfluidic chips utilized the self-organized vascularization method to establish microvascular networks, by controlling the fluid gradient, changing the interstitial flow rate, or changing the concentration of growth factors [19,20]. Wang et al. [21] successfully constructed a perfusable microvascular model of artery-microvascular networks-vein on a single-layer microfluidic chip using the microporous method. Yu et al. [22] achieved the construction of a series of microvascularized tissues on a single-layer microfluidic chip using a sequential edge-guided method based on spontaneous capillary action. Winkelman et al. developed a cerebral microvascular network model on a single-layer microfluidic chip using a microcolumn method. Interstitial flow has been identified as a crucial factor affecting microvascular growth [5]. However, on single-layer chips, the culture medium channels and tissue chambers were often located in the same layer, limiting the ability to study the impact of various fluidic factors on vascular growth. As a result, many researchers have extended the functional layers of single-layer vascularized chips vertically, exploring chips with multilayer structures. Sun et al. [23] divided the microfluidic channels and tissue chambers on a single-layer chip into 2 different layers, successfully constructing a vascularization model on a double-layer chip. However, the effect of fluid factors on vessel growth in their work needed to be further investigated. In our previous work, the large-scale perfusable microvascular tissue construction on a bilayer chip was achieved, although the preparation process for capillary bursting valves was relatively complex [24]. To simplify the fabrication process of multilayer vascularized chips and expand the fluidic environment within the chip, the introduction of a new process and a modification in the shape of tissue chambers are necessary. These considerations stem from the broad applicability of porous membranes in other multilayer chips, with the goal of constructing innovative multilayer vascularized chips [25,26].

This paper presents a novel 3-layer microfluidic chip for constructing microvascular tissue models in vitro. This multilayer microfluidic chip used a porous membrane as a tissue interface layer to separate the tissue chambers as well as the culture fluid channels, and successfully patterned the hydrogel. More than 10 days of microvascular network culture was achieved on this chip, and 70-kDa fluorescein isothiocyanate (FITC)-dextran was successfully perfused in the vascular network, demonstrating that the generated microvascular networks had good barrier properties. The design of triangle, rectangle, and inverted triangle tissue chambers enables the creation of multiple fluidic environments within a single microfluidic chip, and preliminarily verifies the important effects of interstitial flow direction as well as shear stress on microvascular growth. This work is expected to provide a platform foundation for biomedical engineering.

## Materials and Methods

### Fabrication of the multilayer microfluidic chip

**Fig. 1.**
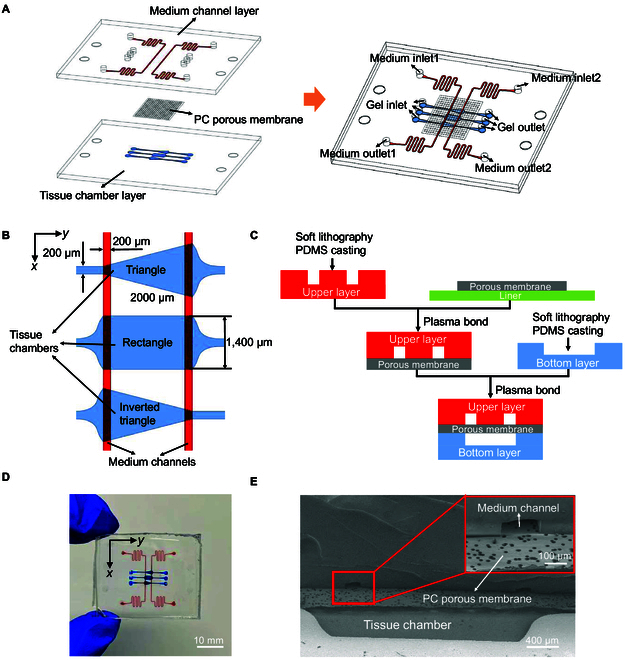
Design and fabrication of the multilayer microfluidic chip. (A) Exploded view and combined view of the multilayer microfluidic chip. (B) Detailed dimensional view of culture medium channels and tissue chambers. (C) Multilayer microfluidic chips fabricated by multiple bonding. (D) Physical diagram of the multilayer microfluidic chip, where red indicated the culture medium channels and blue indicated the tissue chambers. (E) Scanning electron microscope (SEM) image of the culture medium channels–porous membrane–tissue chambers.

As shown in Fig. 1A, the multilayer microfluidic chip consists of the culture medium channel layer at the top, the porous membrane layer in the middle, and the tissue chamber layer at the bottom. The culture medium channel layer and the tissue chamber layer are polydimethylsiloxane (PDMS, Dow Corning) blocks, and the porous membrane layer is the polycarbonate (PC, Sterlitech) filter membrane with 20 μm pore size. It was fabricated through conventional soft lithography and replica molding. The detailed configuration and the fabrication process of multilayer microfluidic chip are given in Fig. S1.

Firstly, we used AutoCAD to design 2-dimensional microchannel graphics of medium channel layer and tissue chamber layer. Then, we fabricated the images into film mask version. Subsequently, a 100-μm-thick SU-8 2050 photoresist was photoengraved on the silicon wafer according to the microchannel pattern of the mask plate. After treatment with propylene glycol methyl ether acetate, it is used in the master mold.

The PDMS base mixed with a curing agent at a ratio of 10:1 (w/w) was poured on the silicon wafer and cured for 2 h in a 65°C dry oven. After the cured PDMS was peeled off from the silicon wafer, perfusion ports for culture medium and hydrogel gel were punched out at the culture medium channel layer using a 1-mm biopsy punch. Commercially available PC membranes were cut to the desired shape using surgical scissors. As shown in Fig. 1C, the multilayer microfluidic chip was bonded multiple times in a certain order after oxygen plasma treatment [27]. Four glass tubes bonded with uncured PDMS to the inlet and outlet of the culture medium channel were used to store the culture medium. The chip was placed in a 65°C desiccator for at least 2 h to maintain the seal and sterilized by UV light for at least 30 min before each experiment.

### Cell culture

Human umbilical vein endothelial cells (HUVECs) were purchased from Lonza and cultured in gelatin-coated (0.5%) dishes using endothelial growth medium (EGM-2, Lonza). HUVECs were transfected with lentivirus to express red fluorescent protein (RFP) and were used after being cultured to passages 4 to 7. Normal human lung fibroblasts (NHLFs) were purchased from Lonza, cultured in fibroblast growth medium (FGM, Lonza), and used after culturing to passages 4 to 8. Growth factors secreted by NHLFs promote HUVECs sprouting and proliferation, ultimately leading to angiogenesis. The cultured HUVECs and NHLFs were removed from the culture dishes using 0.25% trypsin–EDTA (Gibco). All cells were cultured in a humidified incubator (Thermo Fisher Scientific) at 37 °C and 5% CO_2_.

### Gel patterning and cell culture on the multilayer microfluidic chip

The process of gel patterning and cell culture on the multilayer microfluidic chip included preparation of the cell–gel mixture, injection of the cell–gel mixture into the chip, and injection of the culture medium into the chip. The entire process must be completed within 90 min. First, after collecting the cells from the dishes, RFP-HUVECs (concentration of the 7×10^6^ cells/ml) and NHLFs (concentration of the 7×10^6^ cells/ml) were suspended in fibrinogen solution (10 mg/ml, Sigma-Aldrich) at a ratio of 1:1. Second, the cell-fibrinogen suspension was mixed with 50 U/ml thrombin (Sigma-Aldrich) to a final concentration of 3 U/ml, and then the mixture was rapidly injected into the tissue chamber through the gel loading port using a micropipette. The gel was allowed to polymerize in an incubator at 37 °C for 10 min. Third, EGM-2 (Lonza) medium was passed into the medium channel as shown in Fig. S2, and the reservoirs were filled to different medium heights (Inlet 1: 25.5 mm H_2_O, Inlet 2: 15.5 mm H_2_O, Outlet 1: 12.5 mm H_2_O, Outlet 2: 2.5 mm H_2_O). Initially, the hydrostatic pressure of approximately 10 mm H_2_O was applied to the left and right sides of the tissue chamber, resulting in a continuous unidirectional flow of interstitial fluid from left to right, which was sufficient to induce microvascular network sprouting. To simulate the unidirectional flow of human blood, the medium was refilled to the initial condition every 24 h to produce continuous interstitial flow, and the microvascular networks were cultured for 10 days or longer.

### Finite element simulation

In this work, COMSOL Multiphysics was used for finite element simulation. Since the density change of the simulated fluid is very small, the heat caused by viscous dissipation is ignored and the fluid is assumed to be a Newtonian fluid; the incompressible Navier–Stokes and continuity equations were used to simulate fluid flow in capillaries, as described below:

$$\begin{array}{c} \rho \left[\frac{\partial u}{\partial t}+\left(u\cdot \nabla \right)u\right]=-\nabla \cdot p+\nabla \cdot \left[\mu \left(\nabla u+u^{T}\right)\right]+F \end{array}$$ (1)

∇·u=0 (2)

where *p* is the pressure, *u* is the fluid velocity field, *t* is the time, ρ is the density, *μ* is the viscosity, and **F** is the term representing interfacial tension.

Since hydrogels can often be used as porous media materials after solidifying in tissue chambers, the process of stimulating cell and tissue growth of culture solution in hydrogels can be regarded as the flow of fluid in porous media. Therefore, we choose Darcy’s law to study.

Darcy’s law is one of the basic formulas to reveal the law of porous media flow in fluid, which reveals the close relationship between the rate of fluid passing through porous media and the pressure gradient, as described below:

$$q=-\frac{k}{\mu }\nabla p$$ (3)

where *q* is the flow vector per unit area, μ is the fluid viscosity, *k* is the permeability, and p is the pressure. This formula clearly shows the close relationship between the flow rate per unit area and the pressure gradient, according to which we can infer the distribution of the velocity field and the pressure field of the fluid in the porous medium.

Finite element simulation of the fluid profile through the tissue chamber ECM was performed using the Free and Porous Media Flow module in COMSOL. Based on previous works [28,29], the permeability of the gel was set to 1.5 × 10^−13^ m^2^, the porosity was set to 0.99, the dynamic viscosity was set to 0.79 × 10^−3^ Pa·s, and the hydrostatic pressure on both sides of the tissue chamber was set to 10 mm H_2_O. When simulating fluid conditions in multilayer chips, we construct a physical field control grid in all units of the 3D model, forming 632,094 tetrahedrons with a total of 923,980 cells and an average unit mass of 0.5882. Satisfy the mesh quality of 3D model. The fully coupled linear solver used is the algebraic multigrid, which avoids the hassle of creating rough grids for complex geometries with small details. The relative tolerance is 0.001. Since what we simulate in this physical field is the process of the chip in a steady state, there is no simulation time step.

In order to accurately simulate the flow within the lumen of microvessels perfused with dextran, we first used FIJI (ImageJ) to binarize the red fluorescence images of microvessels and extract the structure of the microvascular network (the specific operations are as follows: first, the image is converted to 8-bit grayscale, then run Image→Adjust→Threshold to adjust the threshold and apply). The binarized images were then converted to DXF files using Img2CAD. The DXF file was imported into COMSOL for analysis. The incompressible Navier–Stokes equations were calculated using the laminar flow module in COMSOL. The density of the dextran solution was set to 1,000 kg/m^3^, and the dynamic viscosity was set to 1×10^−3^ Pa·s. The pressure at the left inlet of the microvascular network was set to 10 Pa, and the pressure at the right outlet was set to 0 Pa. We also construct a physical field control grid in all cells of the 3D model, forming 20,773 network vertices with a total of 33,405 units and an average cell mass of 0.7722. The quality of the cell mesh is high. The fully coupled linear solver used is the Direct Solver, and the relative tolerance is 0.001.

### FITC-dextran perfusion

To verify the perfusion capacity of the microvascular networks of this chip based on a porous membrane configuration under different flow conditions, FITC-dextran perfusion tests were performed [24,28]. Firstly, DPBS with the same hydrostatic pressure profile as the culture medium was utilized instead of the culture medium and the chip was placed on a microscope carrier stage. Then, 70-kDa FITC-dextran (final concentration of 50 μg/ml, Sigma-Aldrich) was added to the hydrostatic pressure-measured medium reservoir to observe whether the 70-kDa FITC-dextran solution could be perfused into the microvascular networks through the medium channels.

### Data collection and statistical analysis

Bright-field and fluorescence images were taken by an inverted microscope (Eclipse Ti2, Nikon) with a fluorescence system. Chip cross-section image was taken by a SEM (Regulus8230, Hitachi). Each vascularization experiment was conducted by at least 4 independent tests. The images were preprocessed using FIJI (ImageJ), and the number of junctions, vessel length, and percentage of vessel area were calculated using AngioTool (NIH). The data obtained above were analyzed and presented in graphical form using Microsoft Excel and Origin. Data were shown as mean ± standard deviation (SD). Statistical comparisons of analyzed values were obtained from unpaired 2-tailed Student’s *t* tests with *P* value thresholds set at *P < 0.05 for statistical significance (**P < 0.01; ***P < 0.001) and *P* value thresholds set at *P* > 0.05 for not significant (ns).

## Results

### Design principles of the multilayer vascularized microfluidic chip

In the past works [30,31], it was possible to generate microvascular tissues in single-layer microfluidic chips by using biological self-assembly methods. However, the micropillar structures were usually set up between the culture medium channels and the tissue chambers in the single-layer microfluidic chip. The setup of the permanent barrier led to the restriction of the microfluidic modulation ability, as well as the great spatial limitation of the microvascular tissue growth morphology. Arslan et al. developed a fully vascularized and perfusable cardiac microtissue chip. This chip features a rectangular cell chamber and 2 cultivation channels, allowing for the adjustment of culture medium to apply different static pressure stimuli for angiogenesis. However, the microcolumn structure faces challenges in effectively filling the chamber with the cell–gel mixture [32]. Sun et al. [23] developed a rhomboidal vascularized organ chip, which can realize the use of serpentine pipes to reduce liquid storage tanks, but at the same time, it also fixed the pressure difference of the culture flow path. A capillary burst valve was set to reduce the pressure in the tissue chamber, which could not avoid the leakage of hydrogel from the microcolumn into the culture fluid flow path during injection. The chip functions of the above two are limited, and it is impossible to carry out further research in many aspects. Therefore, it was necessary to arrange the different functional fluidic modules in layers. In this work, a porous membrane was introduced between the upper and bottom PDMS layers to separate the chambers, forming a sandwich-type multilayer microfluidic chip with “culture medium channels–porous membrane–tissue chambers” from top to bottom, as shown in Fig. 1A. It is reported that the porous membrane structure successfully realized the patterning of hydrogels containing hepatocytes in the multilayer microfluidic chip [33]. In this work, the porous membrane design in the middle prevented the culture medium channel from being blocked when the hydrogel with cells was injected, and also ensured the effective stimulation of the endothelial cell-filled tissue chambers by the culture medium through the porous membrane. The commercially available 20-μm pore size PC filter membrane met the above requirements. Fig. S3 describes the principle of using the capillary action to confine the gel within the tissue chambers in the multilayer microfluidic chip containing the porous membrane [28].

The single-layer microfluidic chip was characterized by its ability to alter the pressure gradient on both sides of the fluid channel to provide appropriate flow rate stimulation, thereby inducing endothelial cells to sprout and generate microvessels [15]. As shown in Fig. 1B, in the sandwich-type multilayer microfluidic chip design, the culture medium channel layer consisted of left and right culture medium channels separated by 2 mm. Due to the presence of the porous membrane, the overall width of the culture liquid channel could be designed to be 200 μm. A variety of different fluid characteristics could be generated on the single microfluidic chip by varying the shape of the tissue chambers, such as flow velocity and shear stress. The tissue chambers were designed with 3 different shapes, including triangle, rectangle, and inverted triangle, when viewed from left to right. The length of all tissue chambers from left to right was set at 2 mm. The width of the rectangle chamber was 1,400 μm. The width of the left side of the triangle chamber was 200 μm, and the right side was 1,400 μm. The width of the left side of the inverted triangle chamber was 1,400 μm, and the right side was 200 μm, which was the opposite of the triangle chamber. The height of the culture medium channels and tissue chambers was set at 100 μm. Furthermore, a PC membrane with a pore size of approximately 20 μm, a thickness of around 12 μm, and a porosity of 12% to 15% was chosen. Fig. 1D shows the multilayer microfluidic chip after multiple bonding, in which the red channels were culture medium channels and the blue channels were 3 different shapes of tissue chambers. Fig. 1E shows the scanning electron microscope (SEM) cross-sectional image of the multilayer microfluidic chip, where it could be observed that the PC porous membrane with uniformly distributed pores separated the culture medium channels from the tissue chambers.

The blood microcirculation in the human body was a unidirectional flow process from arterioles to capillaries and then to veins, and the microcirculation model of artery–capillary–vein in the human body could be simulated by regulating the hydrostatic pressure difference between the left and right culture medium channels.

### Finite element simulation of interstitial flow

**Fig. 2.**
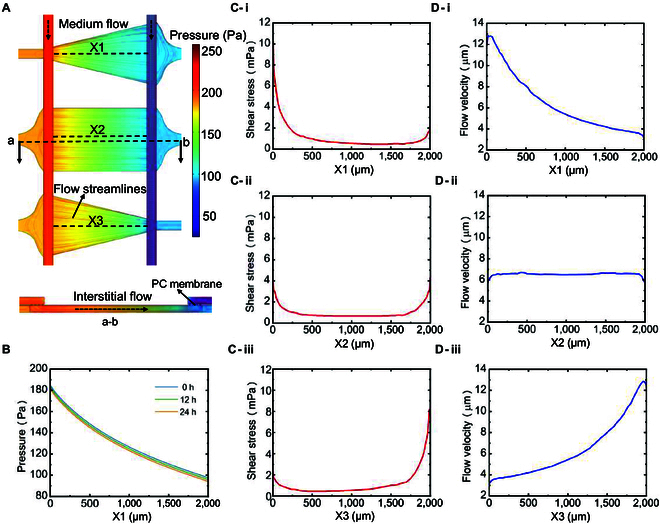
Simulation of fluid distribution inside the tissue chambers. (A) Pressure gradient distributions and flow directions for 3 shapes of tissue chambers, including triangle, rectangle, and inverted triangle tissue chambers. (B) Simulation results of the pressure gradient distribution during a fluid exchange period (*t* = 0, 12, and 24 h). (C) Simulation results of shear stress along (i) X1, (ii) X2, and (iii) X3 profile lines. (D) Simulation results of flow velocity along (i) X1, (ii) X2, and (iii) X3 profile lines.

Fig. 2A shows the hydrostatic pressure distribution in 3 different shapes of tissue chambers at the moment of 24 h. The red lines represent the flow lines in the tissue chambers, and the rainbow-colored gradients indicate the hydrostatic pressure distributions in the culture fluid channels and tissue chambers. By analyzing the profile lines in the center of the 3 tissue chambers (X1, X2, and X3), the interstitial flow in each tissue chamber can be assessed. Taking the triangle tissue chambers as an example, Fig. 2B shows the hydrostatic pressure distribution profiles in the triangle tissue chambers at the moments of 0, 12, and 24 h, indicating that the hydrostatic pressure drop in each tissue chamber from left to right basically remained around 90 Pa at different moments during the 24-h period at all times.

As shown in Fig. 2C-i, in the triangle tissue chamber, the shear stress of the interstitial flow reached a maximum value at the left end (~9 mPa), decreased to a minimum value in the middle region (~0.3 mPa), and gradually increased again at the right end (~2 mPa). As shown in Fig. 2C-ii, in the rectangle tissue chamber, the shear stress of the interstitial flow had higher values at the left and right ends (~4 mPa) than at the middle region (~0.4 mPa). As shown in Fig. 2C-iii, in the inverted triangle tissue chamber, the shear stress of the interstitial flow had lower values at the left end (~2 mPa) to the middle (~0.3 mPa), and then gradually increased toward the right end to the maximum value (~8 mPa). Among these chamber shapes, the rectangle chamber had the most uniform distribution of shear stress and the lowest mean value. The shear stress profiles of triangle and inverted triangle tissue chambers showed opposite distribution characteristics. The general pattern showed a lower shear stress where the chamber width was large and a higher shear stress where the chamber width was small.

As shown in Fig. 2D-i, -ii, and -iii, the distribution of shear stress generated by the interstitial flow showed a positive correlation with that of the flow velocity in the same shaped tissue chambers. According to the simulation results, the flow velocity range of all tissue chambers was between 3 and 13 μm/s, which could provide sufficient stimulation for endothelial cell germination to promote microvessel generation [34]. The different shapes of the tissue chambers had different characteristics of interstitial flow distribution, which could provide the fluid factor explanation for the different characteristics of microvessel networks generated in each tissue chamber in the subsequent vascularization experiments.

By taking into account the shear stress and the direction of interstitial flow in various chamber shapes, which may affect the length, density, and number of connections in the vascular morphology, it is possible to culture a network of microvessels with different vascular morphology characteristics in a single microfluidic chip.

### Generation of microvascular networks of multiple shapes

**Fig. 3.**
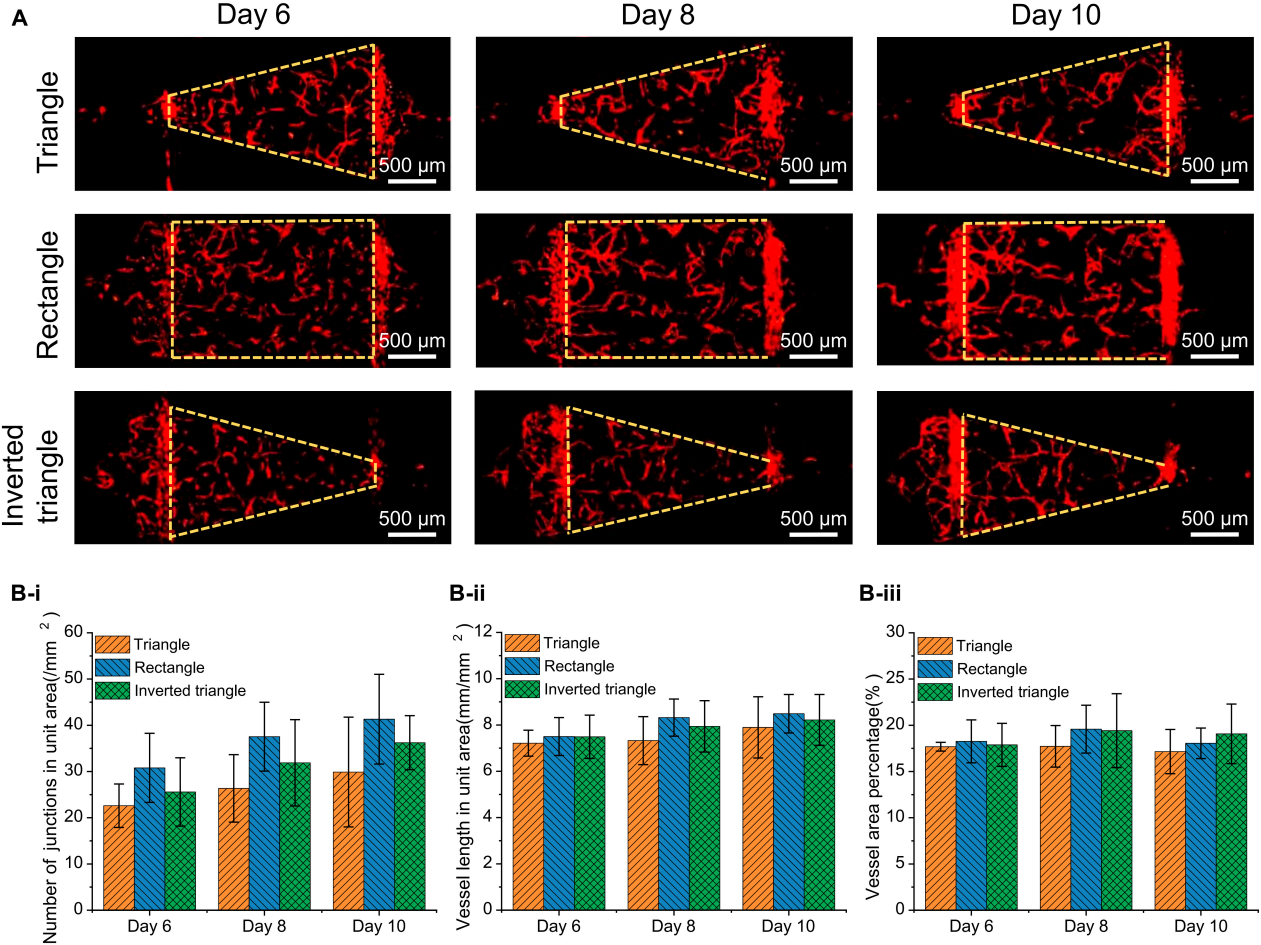
Generation of multishape microvascular networks in multilayer microfluidic chips. (A) Microvascular networks were generated from Day 6 to Day 10 in 3 shapes of tissue chambers, including triangle, rectangle, and inverted triangle tissue chambers. (B) Quantitative analysis of the (i) number of junctions in unit area, (ii) vessel length in unit area, and (iii) vessel area percentage in the 3 chambers on Day 6, Day 8, and Day 10 (mean ± SD, *n* = 4).

Fig. 3A displays the microvessel images of RFP-HUVECs in 3 different shaped chambers on Day 6, Day 8, and Day 10. In each chamber, microvessel fragments showed gradual growth on Day 6. By Day 8, these fragments began to connect, forming microvessel networks with thickened vascular lumens. By Day 10, the area of interconnected microvessels continued to increase, forming a fully connected network locally.

As shown in Fig. 3B, the number of junctions and vessel length exhibited a growth trend from Day 6 to Day 10, indicating positive growth of the microvessels. The area percentage of microvessels in all chambers ranged from 17% to 20%, showcasing high throughput and reproducibility of microvascular tissue generation in the multilayer microfluidic chip.

Remarkably, the rectangle chamber produced the densest microvascular networks. Additionally, this chamber exhibited the most uniform distribution of interstitial flow shear stress with the lowest mean value. Previous studies indicate that lower shear stress can enhance microvessels sprouting and the formation of denser microvascular networks. The morphology of the densest microvascular networks in the rectangle chamber was associated with lower shear stress [35].

### Interstitial flow guides endothelial cell migration and growth during angiogenesis

**Fig. 4.**
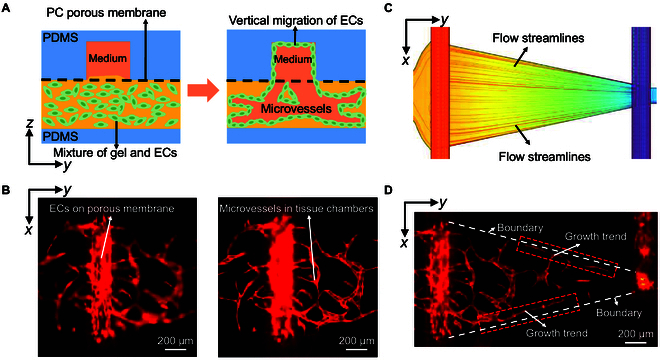
Fluid stimulation leads to vertical migration of endothelial cells and influences the direction of microvascular growth. (A) Schematic diagram of vertical migration of endothelial cells. (B) Vertical migration of endothelial cells stimulated by a gradient of culture growth factors grows into the culture channels. (C) Simulated flow line diagram of an inverted triangle tissue chamber. (D) Microvascular networks grow along the boundary of tissue lumen boundaries under the influence of flow streamlines.

Fig. 4A illustrates the schematic diagram of endothelial cells generating blood vessels under continuous flow stimulation in the culture medium within a multilayer microfluidic chip. In this process, endothelial cells grow on the porous membrane and migrate into the culture medium channel. As shown in Fig. 4B, RFP-HUVECs migrated vertically through the porous membrane toward the direction of the culture medium channel. This migration may be stimulated by the gradient of growth factors in the culture medium, causing endothelial cells to migrate in the opposite direction along the culture medium channel flow [36]. It should be noted that the culture medium channels and tissue chambers in the multilayer microfluidic chip were not on the same layer, but separated by 2 independent layers of PDMS blocks through the porous membrane. By adjusting the focal plane of the microscope, the distance between the porous membrane plane of the culture medium channels and the microvascular plane of the tissue chambers could be observed to be approximately 100 μm, consistent with the thickness of the tissue chamber layer being 100 μm.

As shown in Fig. 4C and D, in one of the inverted triangle tissue chambers, the microvessel growth direction of the upper and bottom boundaries was consistent with the direction of the boundary flow lines obtained from the simulation. This suggested that the growth of microvessels was guided by the fluid, which promoted the growth of microvessels in the direction of flow. Movie S1 demonstrated the presence of cells within the microvascular lumen under bright-field observation as the fluid flowed through, suggesting that the microvessels had acquired more realistic physiological structural characteristics with closed lumens. In conclusion, the above phenomena reveal that fluids play an important role in directing microvascular growth.

### Interstitial flow direction and shear stress act on angiogenesis

**Fig. 5.**
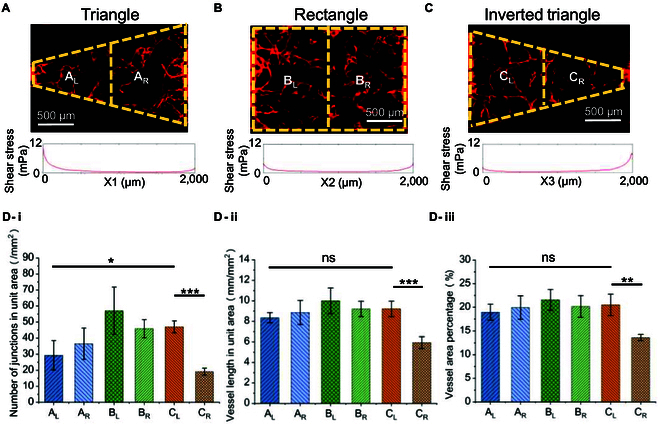
Quantitative analysis of microvascular parameters in the left and right half regions of 3 shaped chambers. (A) Schematic diagram of the left half (A_L_) and right half (A_R_) regions in the triangle tissue chamber. (B) Schematic diagram of the left half (B_L_) and right half (B_R_) regions in the rectangle chamber. (C) Schematic diagram of the left half (C_L_) and right half (C_R_) regions in the inverted triangle chamber. (D) Quantitative analysis of (i) number of junctions in unit area, (ii) vessel length in unit area, and (iii) vessel area percentage in each region (mean ± SD, *n* = 4).

In order to analyze the shear stress of different shapes of interstitial flow in combination with microvascular growth characteristics. As shown in Fig. 5A to C, 3 different shapes of tissue chambers cultured up to Day 10 were divided into left and right equal-width parts for microvascular network characterization. This included the left (A_L_) and right (A_R_) halves of the triangle chamber, the left (B_L_) and right (B_R_) halves of the rectangle chamber, and the left (C_L_) and right (C_R_) halves of the inverted triangle chamber.

As shown in Fig. 5D-i, in the triangle chamber, the average shear stress in the A_L_ region was markedly higher than in the A_R_ region. The parameters in the A_L_ region (number of junctions in unit area: 29.38 mm^−2^, vessel length in unit area: 8.36 mm∙mm^−2^, vessel area percentage: 18.96%) were all lower than those in the A_R_ region (number of junctions in unit area: 36.55 mm^−2^, vessel length in unit area: 8.86 mm/mm^2^, vessel area percentage: 19.94%), but the differences were not marked. Overall, the differences in vascular characteristics between the A_L_ and A_R_ regions were consistent with the lower shear stress promoting microvascular growth and sprouting [35,37].

As shown in Fig. 5D-ii, in the rectangle chamber, the shear stress distribution was more uniform, with the lowest average shear stress. The parameters in the B_L_ region (number of junctions in unit area: 57.09 mm^−2^, vessel length in unit area: 10 mm/mm^2^, vessel area percentage: 21.58%) were all higher than those in the B_R_ region (number of junctions in unit area: 45.91 mm^−2^, vessel length in unit area: 9.21 mm/mm^2^, vessel area percentage: 20.18%). It has been reported that the direction opposite to the interstitial flow promotes vessel sprouting, while the direction opposite to the flow inhibits vessel sprouting. The vascular characteristics in the rectangle chamber may be related to the concentration distribution of growth factors and the growth of the microvascular network against the culture medium flow direction [36].

As shown in Fig. 5D-iii, in the inverted triangle chamber, the average shear stress in the C_L_ region was significantly lower than that in the C_R_ region. The parameters in the C_L_ region (number of junctions in unit area: 47.03 mm^−2^, vessel length in unit area: 9.22 mm/mm^2^, vessel area percentage: 20.52%) were all higher than those in the C_R_ region (number of junctions in unit area: 19.16 mm^−2^, vessel length in unit area: 5.93 mm/mm^2^, vessel area percentage: 13.62%). Morphologically, the microvascular network in the C_L_ region was denser, while the C_R_ region only exhibited vascular fragments. This suggests that under prolonged high shear stress stimulus, the microvascular network undergoes significant degradation [35,37].

Additionally, when comparing the A_L_ region and the C_L_ region, the number of junctions in unit area (29.38 mm^−2^) in the A_L_ region was significantly lower than that in the C_L_ region (47.03 mm^−2^), but the differences in other indicators were not significant. This is consistent with the notion in the literature that higher shear stress weakens the ability for vessel sprouting and formation of channels [35].

According to the experimental results in Fig. 5, the microvascular index parameters of the rectangular chamber were better than those of the other 2 chambers, and the angiogenesis was more average. This is related to the lower average shear stress. In the triangular chamber, more blood vessels were formed at the lower shear stress. The blood vessels at both ends of each chamber were relatively dense, because we infiltrated with collagen in the channel, and the fluorescent cells tended to grow toward the flow channel.

In general, the simulation results are in good agreement with the experimental results. In this multilayer microfluidic chip, the important effects of factors such as fluid flow direction and shear stress on microvascular growth were preliminarily verified, suggesting that this multilayer microfluidic chip provides a potential engineering tool for investigating the effects of fluidic factors on microvessels. Further exploration is needed in order to elucidate the specific mechanism of shear stress on blood vessel growth at the microscale level.

### Perfusion capacity of the microvascular networks

As shown in Fig. 5A, after about 5 min of perfusion with 70-kDa FITC-dextran solution, the continuous flow of dextran solution appeared in the microvessels of the inverted triangle tissue chamber on Day 12, indicating that the generated microvascular networks had good connectivity and closed lumen structure. However, it should be noted that the perfusion ability of the microvascular networks was also affected by various factors such as the type of endothelial cells and the structure of the microfluidic chips. Further studies are needed to ensure the stability of the generated microvascular networks with good perfusion ability.

**Fig. 6.**
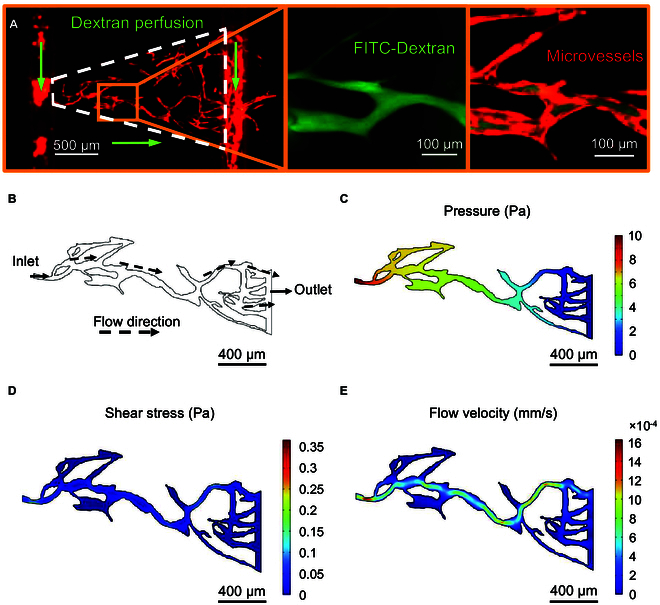
Perfusion of FITC-dextran in microvessels and simulation results of fluid pressure, shear stress, and flow velocity distribution inside microvessels. (A) FITC-dextran successfully perfused microvessels without marked leakage. (B) Microvessel profiles extracted by image processing. (C) Hydrostatic pressure distribution inside the microvessels. (D) Shear stress distribution inside microvessels. (E) Flow velocity distribution inside the microvessels.

To further study the microenvironment of engineered microvascular networks, simulations were conducted on the flow velocity and shear stress within the vascular lumens. Fig. 6B to E shows the simulated results of successfully perfused microvessel profiles and the pressure, flow velocity, and shear stress within the microvessels. The maximum and average flow velocities inside the microvessels obtained from simulations were 1.6259 and 0.2033 mm/s, respectively. The maximum and average shear stresses along the lumen walls were 3.7 and 0.15 dyn/cm^2^, respectively. These values were consistent with the physiological features of blood flow in certain capillary lumens of the body [38–40].

## Discussion

In previous designs, organ-on-a-chip devices were typically fabricated with 2 layers, where only the channel layer was responsible for cell culture and nutrition supply. A porous membrane was added to separate the culture medium channels from the tissue chambers. This innovation not only increased the volume of the culture chambers compared to single-channel vascularized microfluidic chips but also allowed for different feeding strategies of the culture medium. The single-channel-layer chips faced risks such as cell leakage through micropillars into the culture channels when injecting cell–gel mixture into the chambers. Moreover, culture channels could only be designed around the tissue chambers, with limited directions of shear stress and nutrient supply to cells in the chambers provided horizontally by structures like micropillars. However, separating the culture medium channels from the cell chambers using porous membranes facilitated easier injection of cell gels and allowed more flexible adjustment of the culture medium’s hydrostatic pressure of culture medium reservoirs. Based on our experiments, it shows that with the hydrostatic pressure difference between 2 culture medium channels, there is much higher medium transportation across the PC membrane as well as inside the gel, as shown in Fig. S5. This setup provided not only horizontal shear forces but also vertical ones, enabling real 3-dimensional fluid flow environment, further realizing 3D cell culture.

Reports suggest that, exceeding a certain shear stress threshold in low-shear-stress areas can induce vascular sprouting, promote endothelial cell proliferation and migration, and form new vascular structures, in spite of the diminishment of angiogenesis under high shear stress. By designing triangular and inverted triangular chambers, a microcontroller creates both high and low shear stresses within a tissue chamber [35,37]. By designing triangular and inverted triangular chambers, a microcontroller can create a fluid environment with both high and low shear stresses, validating the phenomenon of low shear stress promoting microvascular growth [36]. Additionally, reports indicate a tendency for microvessels to grow against the flow of culture medium. By configuring unidirectional interstitial flow from left to right and rectangular chambers, the reverse growth of microvessels was verified.

This study designed tissue chambers with different shapes to create varying shear force conditions, resulting in different patterns of blood vessel growth. It preliminarily confirmed the correlation between fluid flow direction, shear stress, and the state of blood vessel growth. Simulated results of fluid flow velocity and shear stress infusion of dextran solution into microvascular chambers are consistent with fluid characteristics of human microvessels, demonstrating that this microfluidic chip has the capability to highly simulate human organ structure and function.

At this stage, we lack data to prove that existing experimental results provide the optimal geometric and fluid parameters. In our future work, firstly, we will focus on the precision of microenvironment simulation and on the complexity of fluid control, to improve the similarity of multilayer microfluidic chips with human internal environment. Besides, appropriate fluid parameters are under exploration, to help comprehensively understand the regulatory role of vascular microenvironments on cell activities. We expect this chip not only to be used for studying the impact of fluid factors on microvascular growth but also to enable coculture of different cells and vascular tissues in chambers, forming a multiorgan system within a single chip through specific connections.

## Conclusion

In conclusion, we present a novel 3-layer microfluidic chip that utilizes a PC porous membrane to efficiently separate the culture medium channels from the tissue lumen. By maintaining the unidirectional flow of culture medium and adjusting the shape of tissue chambers, models of microvascular networks with different characteristics were constructed in 3 different shapes of tissue chambers cultured for more than 10 days, and the important effects of the direction of interstitial flow and shear stress on microvascular growth were preliminarily verified. The microvascular networks cultured for 12 days were successfully perfused with 70 kDa FITC-dextran, which indicated that the generated networks had good barrier properties. The multilayer microfluidic chip is characterized by high throughput as well as the ability to highly mimic human models. In the future, this multilayer microfluidic chip is expected to be an important platform for studying the effects of fluidic factors on microvascular growth.

## Data Availability

The authors confirm that the data supporting the findings of this study are available within the article and its supplementary materials.

## References

[1] Fleischer S, Tavakol DN, Vunjak-Novakovic G. From arteries to capillaries: Approaches to engineering human vasculature. Adv Funct Mater. 2020;30(37):1910811. 33708027 10.1002/adfm.201910811 PMC7942836

[2] Kaully T, Kaufman-Francis K, Lesman A, Levenberg S. Vascularization—The conduit to viable engineered tissues. Tissue Eng Part B Rev. 2009;15(2):159–169. 19309238 10.1089/ten.teb.2008.0193

[3] Kim S-J, Kim M-G, Kim J, Jeon JS, Park J, Yi H-G. Bioprinting methods for fabricating in vitro tubular blood vessel models. Cyborg Bionic Syst. 2023;4:0043. 37533545 10.34133/cbsystems.0043 PMC10393580

[4] Jubelin C, Muñoz-Garcia J, Griscom L, Cochonneau D, Ollivier E, Heymann M-F, Vallette FM, Oliver L, Heymann D. Three-dimensional in vitro culture models in oncology research. Cell Biosci. 2022;12(1):155. 36089610 10.1186/s13578-022-00887-3 PMC9465969

[5] Winkelman MA, Kim DY, Kakarla S, Grath A, Silvia N, Dai G. Interstitial flow enhances the formation, connectivity, and function of 3D brain microvascular networks generated within a microfluidic device. Lab Chip. 2022;22(1):170–192. 10.1039/d1lc00605c PMC9257897 34881385

[6] Yin H, Wang Y, Liu N, Zhong S, Li L, Zhang Q, Liu Z, Yue T. Advances in the model structure of in vitro vascularized organ-on-a-chip. Cyborg Bionic Syst. 2024;5:0107. 10.34133/cbsystems.0107 PMC12063728 40353137

[7] Sun X, Xiang J, Chen R, Geng Z, Wang L, Liu Y, Ji S, Chen H, Li Y, Zhang C, et al. Sweat gland organoids originating from reprogrammed epidermal keratinocytes functionally recapitulated damaged skin. Adv Sci. 2021;8(22):2103079. 10.1002/advs.202103079 PMC8596119 34569165

[8] Lu T, Li Y, Chen T. Techniques for fabrication and construction of three-dimensional scaffolds for tissue engineering. Int J Nanomedicine. 2013;337–350. 23345979 10.2147/IJN.S38635 PMC3551462

[9] Joshi A, Choudhury S, Gugulothu SB, Visweswariah SS, Chatterjee K. Strategies to promote vascularization in 3D printed tissue scaffolds: Trends and challenges. Biomacromolecules. 2022;23(7):2730–2751. 35696326 10.1021/acs.biomac.2c00423

[10] Wang Y, Wang Y, Mei D. Scalable printing of bionic multiscale channel networks through digital light processing-based three-dimensional printing process. 3D Print Addit Manuf. 2020;7(3):115–125. 36655197 10.1089/3dp.2020.0025 PMC9586228

[11] Lee G-h, Huang SA, Aw WY, Rathod ML, Cho C, Ligler FS, Polacheck WJ. Multilayer microfluidic platform for the study of luminal, transmural, and interstitial flow. Biofabrication. 2022;14(2):25007. 10.1088/1758-5090/ac48e5 PMC8867496 34991082

[12] Lee H. Engineering in vitro models: Bioprinting of organoids with artificial intelligence. Cyborg Bionic Syst. 2023;4:0018. 37011281 10.34133/cbsystems.0018 PMC10057937

[13] Li X, Ding W, Wang S, Yang L, Yu Q, Xiao C, Chen G, Zhang L, Guan S, Sun D. Three-dimensional sulfated bacterial cellulose/gelatin composite scaffolds for culturing hepatocytes. Cyborg Bionic Syst. 2023;4:0021. 37223548 10.34133/cbsystems.0021 PMC10202184

[14] Liu H, Wang Y, Cui K, Guo Y, Zhang X, Qin J. Advances in hydrogels in organoids and organs-on-a-chip. Adv Mater. 2019;31(50):1902042. 10.1002/adma.201902042 31282047

[15] Kim S, Lee H, Chung M, Jeon NL. Engineering of functional, perfusable 3D microvascular networks on a chip. Lab Chip. 2013;13(8):1489–1500. 23440068 10.1039/c3lc41320a

[16] Campisi M, Shin Y, Osaki T, Hajal C, Chiono V, Kamm RD. 3D self-organized microvascular model of the human blood-brain barrier with endothelial cells, pericytes and astrocytes. Biomaterials. 2018;180:117–129. 30032046 10.1016/j.biomaterials.2018.07.014 PMC6201194

[17] Nashimoto Y, Okada R, Hanada S, Arima Y, Nishiyama K, Miura T, Yokokawa R. Vascularized cancer on a chip: The effect of perfusion on growth and drug delivery of tumor spheroid. Biomaterials. 2020;229: Article 119547. 31710953 10.1016/j.biomaterials.2019.119547

[18] Niculescu A-G, Chircov C, Bîrcă AC, Grumezescu AM. Fabrication and applications of microfluidic devices: A review. Int J Mol Sci. 2021;22(4):2011. 33670545 10.3390/ijms22042011 PMC7921936

[19] Lee S-R, Kim Y, Kim S, Kim J, Park S, Rhee S, Park D, Lee B, Baek K, Kim H-Y, et al. U-IMPACT: A universal 3D microfluidic cell culture platform. Microsyst Nanoeng. 2022;8(1):126. 36478874 10.1038/s41378-022-00431-w PMC9719897

[20] Quintard C, Tubbs E, Jonsson G, Jiao J, Wang J, Werschler N, Laporte C, Pitaval A, Bah T-S, Pomeranz G, et al. A microfluidic platform integrating functional vascularized organoids-on-chip. Nat Commun. 2024;15(1):1452. 38365780 10.1038/s41467-024-45710-4 PMC10873332

[21] Wang X, Phan DTT, Sobrino A, George SC, Hughes CCW, Lee AP. Engineering anastomosis between living capillary networks and endothelial cell-lined microfluidic channels. Lab Chip. 2016;16(2):282–290. 26616908 10.1039/c5lc01050k PMC4869859

[22] Yu J, Lee S, Song J, Lee S-R, Kim S, Choi H, Kang H, Hwang Y, Hong Y-K, Jeon NL. Perfusable micro-vascularized 3D tissue array for high-throughput vascular phenotypic screening. Nano Converg. 2022;9(1):16. 35394224 10.1186/s40580-022-00306-w PMC8994007

[23] Sun Q, Pei J, Li Q, Chen X, Wang X. Novel microfluidic perfusion bioreactor for vascularized organ-on-a-chip. In: *2019 IEEE/ASME International Conference on Advanced Intelligent Mechatronics (AIM)*. IEEE; 2019. p. 1630–1634.

[24] Yue T, Zhao D, Phan DTT, Wang X, Park JJ, Biviji Z, Hughes CCW, Lee AP. A modular microfluidic system based on a multilayered configuration to generate large-scale perfusable microvascular networks. Microsyst Nanoeng. 2021;7(1):4. 33456784 10.1038/s41378-020-00229-8 PMC7787972

[25] Huh D, Matthews BD, Mammoto A, Montoya-Zavala M, Hsin HY, Ingber DE. Reconstituting organ-level lung functions on a chip. Science. 2010;328(5986):1662–1668. 20576885 10.1126/science.1188302 PMC8335790

[26] Sakai K, Miura S, Sawayama J, Takeuchi S. Membrane-integrated glass chip for two-directional observation of epithelial cells. Sensors Actuators B Chem. 2021;326: Article 128861.

[27] Gopinathan KA, Mishra A, Mutlu BR, Edd JF, Toner M. A microfluidic transistor for automatic control of liquids. Nature. 2023;622(7984):735–741. 37880436 10.1038/s41586-023-06517-3 PMC10600001

[28] Wang D, Li Q, Zhou C, Li Z, Lu K, Liu Y, Xuan L, Wang X. Dissolvable temporary barrier: A novel paradigm for flexible hydrogel patterning in organ-on-a-chip models. Bio-Des Manuf. 2024;7(2):153–166.

[29] Wang X, Phan DTT, Zhao D, George SC, Hughes CCW, Lee AP. An on-chip microfluidic pressure regulator that facilitates reproducible loading of cells and hydrogels into microphysiological system platforms. Lab Chip. 2016;16(5):868–876. 26879519 10.1039/c5lc01563d PMC4911208

[30] Sobrino A, Phan DTT, Datta R, Wang X, Hachey SJ, Romero-López M, Gratton E, Lee AP, George SC, Hughes CCW. 3D microtumors in vitro supported by perfused vascular networks. Sci Rep. 2016;6(1):31589. 27549930 10.1038/srep31589 PMC4994029

[31] Phan DTT, Wang X, Craver BM, Sobrino A, Zhao D, Chen JC, Lee LYN, George SC, Lee AP, Hughes CCW. A vascularized and perfused organ-on-a-chip platform for large-scale drug screening applications. Lab Chip. 2017;17(3):511–520. 28092382 10.1039/c6lc01422d PMC6995340

[32] Arslan U, Brescia M, Meraviglia V, Nahon DM, van Helden RWJ, Stein JM, van den Hil FE, van Meer BJ, Cuenca MV, Mummery CL, et al. Vascularized hiPSC-derived 3D cardiac microtissue on chip. Stem Cell Rep 2023;18(7):1394–1404. 10.1016/j.stemcr.2023.06.001 PMC10362508 37390826

[33] Hegde M, Jindal R, Bhushan A, Bale SS, McCarty WJ, Golberg I, Usta OB, Yarmush ML. Dynamic interplay of flow and collagen stabilizes primary hepatocytes culture in a microfluidic platform. Lab Chip. 2014;14(12):2033–2039. 24770663 10.1039/c4lc00071d PMC4036071

[34] Hsu Y-H, Moya ML, Abiri P, Hughes CCW, George SC, Lee AP. Full range physiological mass transport control in 3D tissue cultures. Lab Chip. 2013;13(1):81–89. 23090158 10.1039/c2lc40787f PMC3510322

[35] Shirure VS, Lezia A, Tao A, Alonzo LF, George SC. Low levels of physiological interstitial flow eliminate morphogen gradients and guide angiogenesis. Angiogenesis. 2017;20:493–504. 28608153 10.1007/s10456-017-9559-4 PMC10597324

[36] Kim S, Chung M, Ahn J, Lee S, Jeon NL. Interstitial flow regulates the angiogenic response and phenotype of endothelial cells in a 3D culture model. Lab Chip. 2016;16(21):4189–4199. 27722679 10.1039/c6lc00910g

[37] Galie PA, Nguyen D-HT, Choi CK, Cohen DM, Janmey PA, Chen CS. Fluid shear stress threshold regulates angiogenic sprouting. Proc Natl Acad Sci USA. 2014;111(22):7968–7973. 24843171 10.1073/pnas.1310842111 PMC4050561

[38] Ivanov KP, Kalinina MK, Levkovich YI. Blood flow velocity in capillaries of brain and muscles and its physiological significance. Microvasc Res. 1981;22(2):143–155. 7321902 10.1016/0026-2862(81)90084-4

[39] Kang H, Bayless KJ, Kaunas R. Fluid shear stress modulates endothelial cell invasion into three-dimensional collagen matrices. Am J Physiol Heart Circ Physiol. 2008;295(5):H2087–H2097. 18805898 10.1152/ajpheart.00281.2008 PMC4747896

[40] Song JW, Munn LL. Fluid forces control endothelial sprouting. Proc Natl Acad Sci USA. 2011;108(37):15342–15347. 21876168 10.1073/pnas.1105316108 PMC3174629

