# Supplementary material for: A Vascularized Multilayer Chip Reveals Shear Stress-Induced Angiogenesis in Diverse Fluid Conditions
Source: Cyborg Bionic Syst. 2025 Feb 28;6:0207. doi: 10.34133/cbsystems.0207 (PMC11870090; doi:10.34133/cbsystems.0207)
Supplement: Supplementary 1 — Figs. S1 to S5 Movie S1 Data S1 [file cbsystems.0207.f1.zip › Supplementary Information.pdf]

Supplementary Information

A vascularized multilayer chip reveals shear stress induced angiogenesis in diverse fluid conditions

Tao Yue[1,2,3,4*], Huiying Yang[1], Yue Wang[2*], Ning Jiang[1], Hongze Yin[1], Xiaoqi Lu[1], Na Liu[1,3,4*] and Yichun Xu[5,6]

[1]School of Mechatronic Engineering and Automation, Shanghai University, Shanghai, China

[2]School of Future Technology, Shanghai University, Shanghai, China

[3]Shanghai Key Laboratory of Intelligent Manufacturing and Robotics, Shanghai University, Shanghai, China

[4]Shanghai Institute of Intelligent Science and Technology, Tongji University, Shanghai, China

[5]National Engineering Research Center for Biochip at Shanghai, Shanghai, China

[6]Shanghai Biochip Corporation (SBC), Shanghai, China

* Corresponding author(s). E-mail(s): Tao Yue (tao_yue@shu.edu.cn)

Yue Wang (yue_wang@shu.edu.cn)

Na Liu (liuna_sia@shu.edu.cn)

Supplementary Figure S1

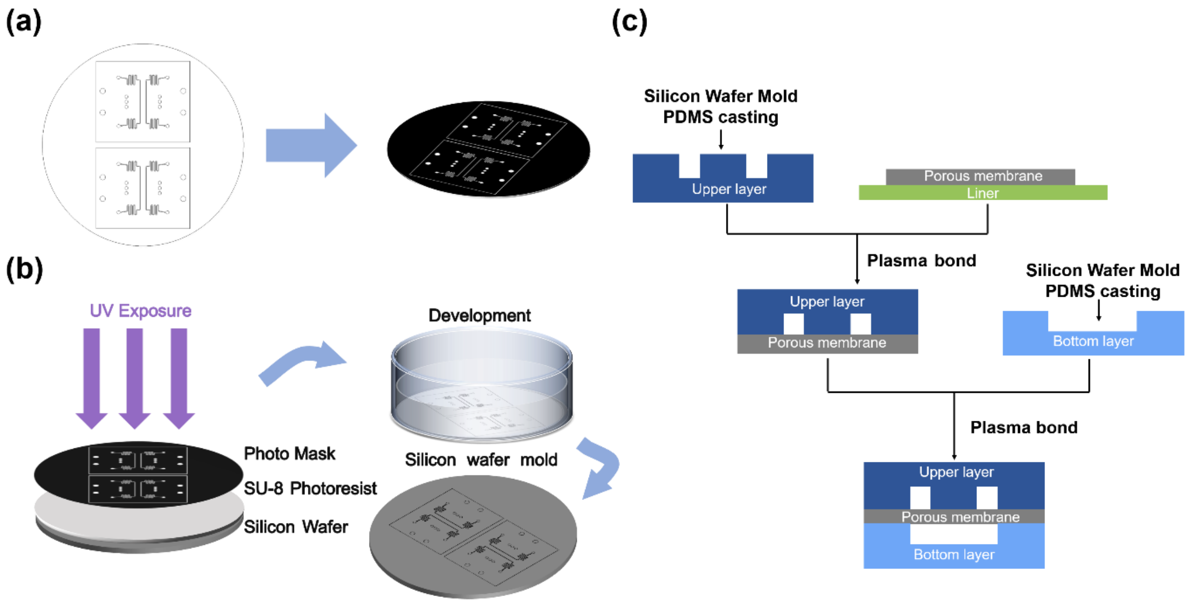

Figure S1 Fabrication of the multilayer microfluidic chip. *(a) Using AutoCAD software to design two-dimensional microchannel graphics. (b) The micropatterns are processed into film mask plates and then made into molds after photoetching into silicon wafers. (c) Multilayer microfluidic chips fabricated by multiple bonding.*

Supplementary Figure S2

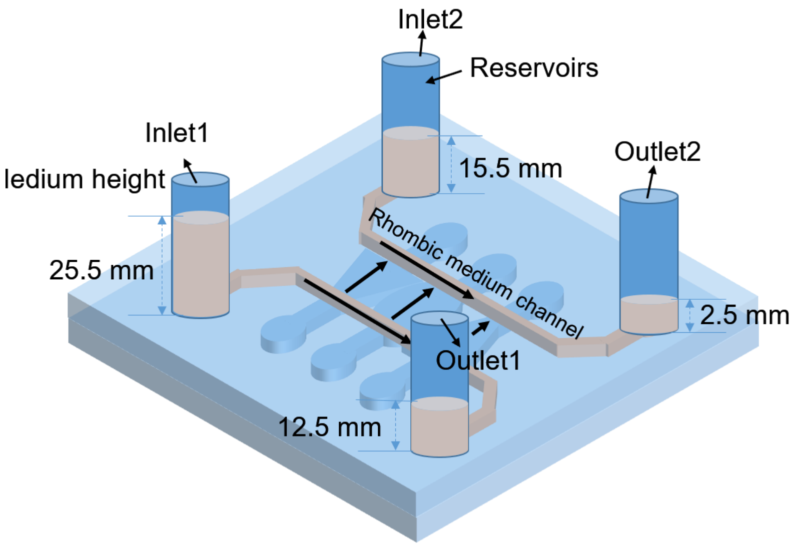

Figure S2 The four reservoirs contained the medium at different heights to generate hydrostatic pressure. *Initially, the height of the culture medium in Inlet1 was 25.5 mm, the height of the culture medium in Inlet2 was 15.5 mm, the height of the culture medium in Outlet1 was 12.5 mm, the height of the culture medium in Outlet2 was 2.5 mm. The medium was refilled to the initial condition every 24 hours to produce continuous interstitial flow.*

Supplementary Figure S3

Figure S3(a) illustrates the equivalent circuit model diagram of the gel loading process in the multilayer microfluidic chip. P_1 and P_2 are the pressure at the inlet and outlet of the gel, respectively, and both of the above can be equated to a DC power supply. This inverted diode represents the micropores of a porous membrane. Its breakdown level corresponds to the breakdown pressure ΔP_{gap}. The resistances R_1 and R_2 are analogous to the hydraulic resistance located at the back and front sides of the microporous-gel interface, respectively. Thus, the pressure P along the micropore-gel interface within the tissue chamber can be defined as follows:

$$P = \frac{R_2}{R_1 + R_2}(P_1 + P_2) - P_2$$

The pressure P increases with externally applied pressure or with the advancement of the gel within the tissue chamber. In order to confine the gel in the porous membrane, ΔP_{gap} and P should satisfy the following relation:

$$P - P_{air} < \Delta P_{gap}$$

Figure S3 (b-d) illustrates the process of a colloid being confined within a flow channel by the surface tension of a porous membrane. where the pore size of the porous membrane is denoted by s and the critical contact angle is denoted by θ_A. According to the Young-Laplace equation, ΔP_{gap} is given by the following equation:

$$\Delta P_{gap} = -2\gamma\left(\frac{\cos\theta_A^*}{s} + \frac{\cos\theta_A}{s}\right)$$

where the new critical forward contact angle $\theta_A^*= \min\{\theta_A+\alpha,\ 180^\circ\}$. Since the gel completely passes through the microporous, $\theta_A^* = 180°$. Based on the property of the microfluidic chip

material, where θ_A≈140°. And the surface tension coefficient of the gel is assumed to be γ=0.072 N/m. Assuming pressure P-P_{air}=3000Pa. When the pore size of the porous membrane is 20, 50, 70 and 100 μm, the corresponding ΔP_{gap} is 12715 Pa、5086 Pa、3633Pa and 2543Pa. Therefore, the use of 20 μm porous membrane can meet the requirements for gel patterning.

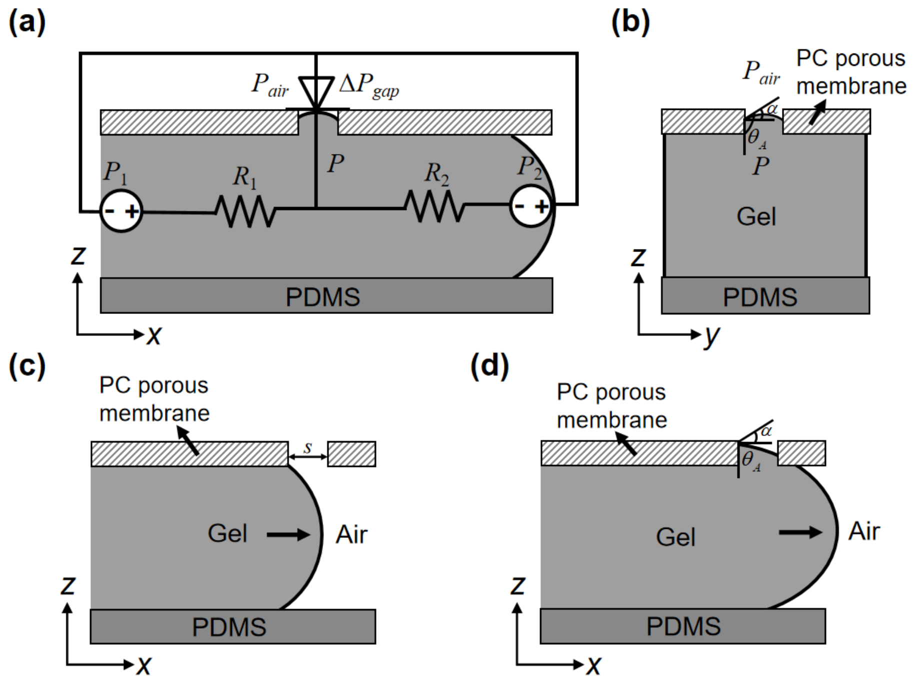

Figure S3 The construction principle of the multilayer microfluidic chip tissue interface containing the porous membrane. *(a) Schematic of the simplified circuit model of the gel loading process. (b) Capillarity at the cross-section micropore. (c) Gel loading before passing through micropore. (d) Gel loading after passing through micropore.*

Supplementary Movie S4 *Movement of cells inside the lumen.*

Supplementary Figure S5

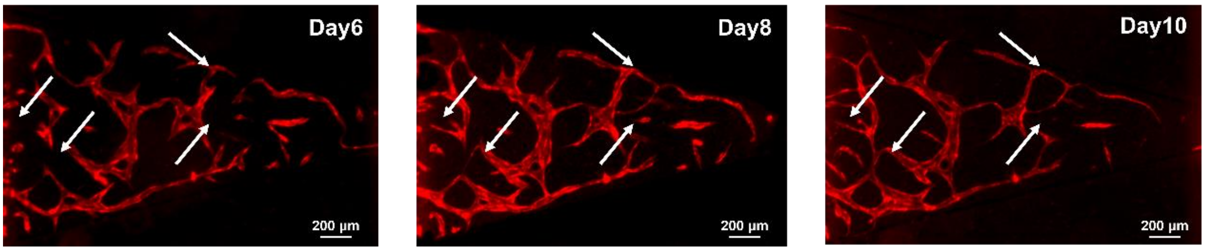

Figure S5 Migration trend of endothelial cells. *The picture is a Mosaic of the images of the inverted triangle chamber on Day 6, Day 8 and Day 10. As time progresses, individual blood vessels gradually connect with each other to form a vascular network. Subsequently, energy distribution within the vessels occurs, leading to the formation of an organized vascular network.*

Supplementary Figure S6

The gravity dominates the medium transportation.

In the gravity-driven group, we added 2000 μL and 1000 μL of filtered red pigment mixed solution at both ends of the two culture channels, along with 1200 μL and 200 μL of pure water. In contrast, the zero gravity-driven group contained 1200 μL and 200 μL of filtered red pigment mixed solution at both ends of the culture channels, along with 1200 μL and 200 μL of pure water. This setup was designed to ensure that both culture channels experienced the same capillary forces conditions during the flow process.

The specific materials used in this experiment are:

1. *The multilayer organ chip used in the paper.*
2. *The hydrogel used to fill the cell chamber: a mixture of fibrinogen and thrombin prepared according to the cell experiment concentration stated in this paper.*
3. *A fluorescent red dye mixed solution diluted 1000 times with pure water, as well as pure water, to replace the original transparent culture medium for easier observation.*

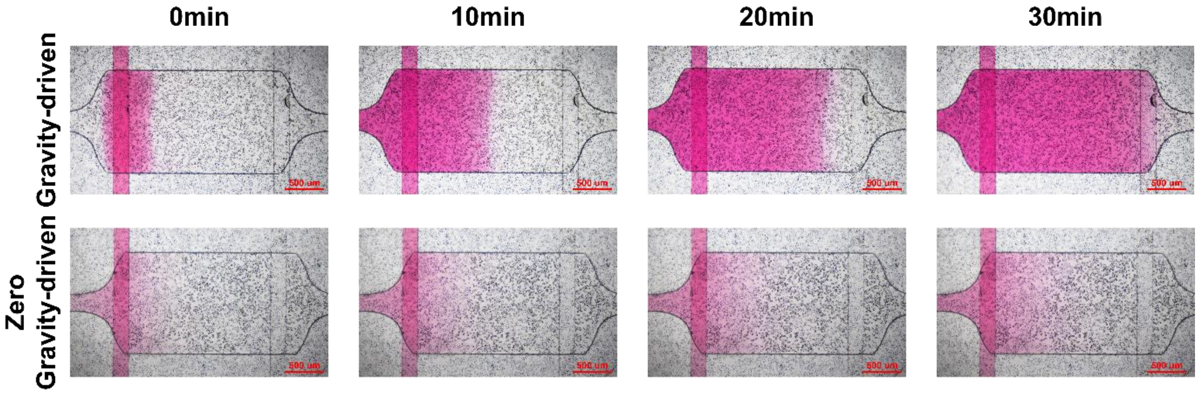

Figure S6 The diffusion variation of the solution inside the Organ-on-chips over time. *Take the same diffusion interface as the starting time and record the diffusion interface every 10 minutes. The gravity-driven group is able to achieve perfusion of the culture medium from left*

to right at around 35 minutes, while in the zero gravity-driven group, the diffusion only reaches nearly half of the chamber at 35 minutes.

Supplementary Excel S*7 The detailed data of* ***Figure 3.***
